# HLA*LA—HLA typing from linearly projected graph alignments

**DOI:** 10.1093/bioinformatics/btz235

**Published:** 2019-04-03

**Authors:** Alexander T Dilthey, Alexander J Mentzer, Raphael Carapito, Clare Cutland, Nezih Cereb, Shabir A Madhi, Arang Rhie, Sergey Koren, Seiamak Bahram, Gil McVean, Adam M Phillippy

**Affiliations:** 1 Institute of Medical Microbiology, University Hospital of Dusseldorf, Dusseldorf, North Rhine-Westphalia, Germany; 2 Genome Informatics Section, Computational and Statistical Genomics Branch, National Human Genome Research Institute, Bethesda, MD, USA; 3 Wellcome Centre for Human Genetics, University of Oxford, Oxford, UK; 4 Big Data Institute, Li Ka Shing Centre for Health Information and Discovery, University of Oxford, Oxford, UK; 5 Laboratoire d’ImmunoRhumatologie Moléculaire, Plateforme GENOMAX, INSERM UMR_S 1109, LabEx TRANSPLANTEX, Fédération de Médecine Translationnelle de Strasbourg (FMTS), Faculté de Médecine, Université de Strasbourg, France; 6 Service d’Immunologie Biologique, Nouvel Hôpital Civil, Strasbourg, France; 7 Medical Research Council: Respiratory and Meningeal Pathogens Research Unit, Faculty of Health Sciences, University of the Witwatersrand, Johannesburg, South Africa; 8 Department of Science/National Research Foundation: Vaccine Preventable Diseases, Faculty of Health Science, University of the Witwatersrand, Johannesburg, South Africa; 9 Histogenetics, Ossining, NY, USA

## Abstract

**Summary:**

HLA*LA implements a new graph alignment model for human leukocyte antigen (HLA) type inference, based on the projection of linear alignments onto a variation graph. It enables accurate HLA type inference from whole-genome (99% accuracy) and whole-exome (93% accuracy) Illumina data; from long-read Oxford Nanopore and Pacific Biosciences data (98% accuracy for whole-genome and targeted data) and from genome assemblies. Computational requirements for a typical sample vary between 0.7 and 14 CPU hours per sample.

**Availability and implementation:**

HLA*LA is implemented in C++ and Perl and freely available as a bioconda package or from https://github.com/DiltheyLab/HLA-LA (GPL v3).

**Supplementary information:**

Supplementary data are available at *Bioinformatics* online.

## 1 Introduction

Genetic variation at the human leukocyte antigen (HLA) loci is associated with many important phenotypes and biological conditions, including autoimmune and infectious disease risk, transplant rejection, and the repertoire of immune-presented peptides (Trowsdale and Knight, 2013). With the growing availability of whole-exome and whole-genome sequencing (WGS) data, the ability to accurately determine the allelic state of the HLA genes (‘HLA typing’) from these data types is becoming increasingly important. The accuracy of standard sequencing data analysis methods in the HLA region is limited by hyperpolymorphism and reference divergence (Dilthey et al., 2015). Specialized HLA analysis methods, however, have been developed (Bai *et al.*, 2014; Dilthey *et al.*, 2016; Huang *et al.*, 2015; Lee and Kourami, 2018; Wittig *et al.*, 2015; Xie *et al.*, 2017); these typically rely on variation-aware alignment approaches, e.g. genome graphs or collections of linear reference sequences. When applied to high-coverage WGS data, the accuracy of these methods can approach that of gold-standard HLA typing assays like sequence-based typing (SBT). HLA type inference from exome or low-coverage WGS data, however, remains more challenging, and most tools do not support long-read data.

We present HLA*LA (‘linear alignments’), a graph-based method with high accuracy on exome and low-coverage WGS data, full support for assembled and unassembled long-read data and a new projection-based approach to graph alignment. Briefly, the alignment process starts with the identification of linear alignments between the input reads and the reference haplotypes that the graph was constructed from; these are projected onto the graph, an approach also used by Lee and Kourami, 2018, and optimized in a stepwise process specific to Population Reference Graphs (PRGs; Dilthey *et al.*, 2015). The intuition behind this is that the (projected) original linear alignment will often be a close approximation to the best graph alignment, except for reads that switch between divergent reference haplotypes. In HLA*LA, these are identified heuristically and trigger a switch into full graph alignment mode. Its hybrid approach enables HLA*LA to often avoid—potentially costly—full graph alignment, to leverage the performance of highly optimized linear alignment algorithms and to carry out graph alignment for both short and long reads in a unified framework.

## 2 Materials and methods

HLA*LA employs a PRG of 13 491 sequences, representing the eight GRCh38 (Schneider *et al.*, 2017) MHC haplotypes and the IMGT exonic and genomic sequences (Robinson *et al.*, 2015). Linear alignments are obtained by aligning input reads against a modified reference genome (GRCh38 plus the eight MHC haplotypes and IMGT genomic sequences) with BWA-MEM (Li, 2013).

Alignments from regions covered by the PRG are projected onto the graph and undergo a three-stage optimization process that draws on the fact that PRGs have, like multiple sequence alignments, a well-defined column structure (‘levels’). We can distinguish between two types of alignment errors: bases aligned to an edge at the wrong level of the graph or bases that are aligned to the right level but the wrong edge. During the first step of the optimization process (‘inspection’), we heuristically identify and remove from the alignment bases which might be aligned to the wrong level of the graph, based on an examination of the alignment’s local gap structure. During the second step (‘polishing’), we efficiently find the highest-scoring graph traversal within the existing level structure of the remaining alignment using dynamic programming. During a final step (‘extension’), the alignment is extended to the complete length of the read in full graph alignment mode; the computational advantage of HLA*LA derives mainly from the fact that this step can be skipped for most reads. For long reads with increased INDEL rates, gaps in the projected alignment are not indicative of potential misalignments; we consequently omit the base removal (during inspection) and re-integration (alignment extension) steps. A full description of alignment projection and optimization for short and long reads is given in Supplementary Note S1.

For HLA type inference, we employ the likelihood model of HLA*PRG (Dilthey *et al.*, 2016). Briefly, at each locus, we maximize $P\left(\text{aligned}\;\text{reads}\right|a_{1},a_{2})$ over all pairs $\left(a_{1},a_{2}\right)$ of possible HLA alleles. For long reads, we increase INDEL rates in the underlying alignment likelihood (Supplementary Note S1).

The complete inference approach of HLA*LA is illustrated in Supplementary Figure S1.

HLA typing of assemblies, which can be used if raw long reads are not available or as a metric of assembly quality, is based on a projection of the GRCh38 MHC reference haplotype annotations onto the assembly. Briefly, MHC-overlapping contigs are identified with nucmer (Delcher *et al.*, 2002). For each identified contig and each reference haplotype (Schneider *et al.*, 2017), a semi-global alignment is computed heuristically (see below); the annotations of the haplotype underlying the highest-scoring alignment are projected onto the contig; HLA gene and exon sequences are extracted based on the projected coordinates. HLA typing is carried out by IMGT database matching (minimum edit distance). The global alignment heuristic uses BWA-MEM for the identification of local alignments (‘diagonals’) between the input sequences; dynamic programming is used to identify the highest-scoring traversal of the global alignment matrix limited to the identified diagonals connected with horizontal or vertical jumps. This approach accounts for different MHC haplotype structures and ensures that each gene is used only once per contig.

## 3 Results

We carry out three experiments (Table 1) to assess the performance of HLA*LA. On high-coverage Illumina WGS data (1000 Genomes Project Consortium *et al.*, 2012; Eberle *et al.*, 2017), HLA*LA achieves a performance of 99.4% averaged over the six classical HLA genes. This is identical to the performance of Kourami and HLA*PRG. xHLA (Xie *et al.*, 2017) achieves an accuracy of 100%, but does not produce calls for *HLA-DQA1*. The performance of HLA*LA under reduced coverage (15×) is relatively stable (90%) and comparable to that of xHLA (91%), where xHLA has higher performance on HLA class I (95% compared to 87%), and HLA*LA has higher performance on HLA class II (94% compared to 84%). Kourami is less stable under reduced coverage (average accuracy 69%). On whole-exome Illumina sequencing data (International HapMap, 2005), HLA*LA outperforms HLA*PRG (93% accuracy compared to 89%) and Kourami at matched call rates (95.3% at 88% call rate compared to 94.6% accuracy at 83% call rate). The overall accuracy of xHLA is 95%, with *HLA-DQA1* remaining uncalled. On whole-genome and targeted PacBio and Nanopore sequencing data (Carapito *et al.*, 2016; Jain *et al.*, 2018; Steinberg *et al.*, 2016), HLA*LA achieves an average accuracy of 98%. Of note, the majority (2034 alleles) of the long-read validation data consists of highly diverse South African samples. HLA*PRG, Kourami and xHLA do not support long-read data. Complete validation results by cohort, a description of the validation cohorts and accessions, and truth HLA types are given in Supplementary Table S1, Supplementary Note S2 and Supplementary Table S2. Computational requirements depend on sample type and coverage (0.65 CPU hours on average for exome samples; between 2.9 and 14 CPU hours for a typical WGS sample; Supplementary Table S3). Assembly typing was successfully applied to assess diploid MHC assembly quality and phasing accuracy in two recent *de novo* assembly projects (Jain *et al.*, 2018; Koren *et al.*, 2018).

**Table 1.** Summary validation results

Cohort	Locus	N	HLA*PRG		Kourami		xHLA		HLA*LA	
			Call rate	Accuracy	Call rate	Accuracy	Call rate	Accuracy	Call rate	Accuracy
Illumina WGS	A	28	1.00	1.00	1.00	0.96	1.00	1.00	1.00	1.00
	B	28	1.00	1.00	1.00	1.00	1.00	1.00	1.00	1.00
	C	28	1.00	1.00	1.00	1.00	1.00	1.00	1.00	1.00
	DQA1	18	1.00	1.00	1.00	1.00	0.00	NA	1.00	1.00
	DQB1	28	1.00	1.00	1.00	1.00	1.00	1.00	1.00	1.00
	DRB1	28	1.00	0.96	1.00	1.00	1.00	1.00	1.00	0.96
Illumina exome	A	58	1.00	0.86	0.76	0.91	1.00	0.98	1.00	0.93
	B	48	1.00	0.85	0.63	0.87	1.00	0.88	1.00	0.92
	C	56	1.00	0.79	0.61	0.97	1.00	0.96	1.00	0.88
	DQA1	58	1.00	0.95	1.00	0.97	0.00	NA	1.00	0.97
	DQB1	58	1.00	0.98	0.97	0.96	1.00	0.95	1.00	0.98
	DRB1	58	1.00	0.91	1.00	0.97	1.00	0.98	1.00	0.91
Long-read WGS	A	6	NA		NA		NA		1.00	1.00
	B	6							1.00	1.00
	C	6							1.00	1.00
	DQA1	6							1.00	1.00
	DQB1	6							1.00	1.00
	DRB1	6							1.00	0.83
Long-read targeted	A	360	NA		NA		NA		1.00	1.00
	B	362							1.00	1.00
	C	362							1.00	1.00
	DQA1	318							1.00	0.97
	DQB1	362							1.00	0.97
	DRB1	280							1.00	0.95

## 4 Conclusion

In summary, HLA*LA improves upon the accuracy of its predecessor HLA*PRG, while being 3–10 times faster and extending HLA typing functionality to long reads and assemblies. HLA*LA is freely available from GitHub and as a bioconda package (Gruning *et al.*, 2018). Generalization of projection-based graph alignment beyond the HLA is a topic for future research.

## Supplementary Material

btz235_Supplementary_Data
