# Supplementary material for: HLA*LA—HLA typing from linearly projected graph alignments
Source: Bioinformatics. 2019 Apr 3;35(21):4394–6. doi: 10.1093/bioinformatics/btz235 (PMC6821427; doi:10.1093/bioinformatics/btz235)
Supplement: btz235_Supplementary_Data [file btz235_supplementary_data.zip › btz235-suppl_data/Supplementary Figure S1.pdf]

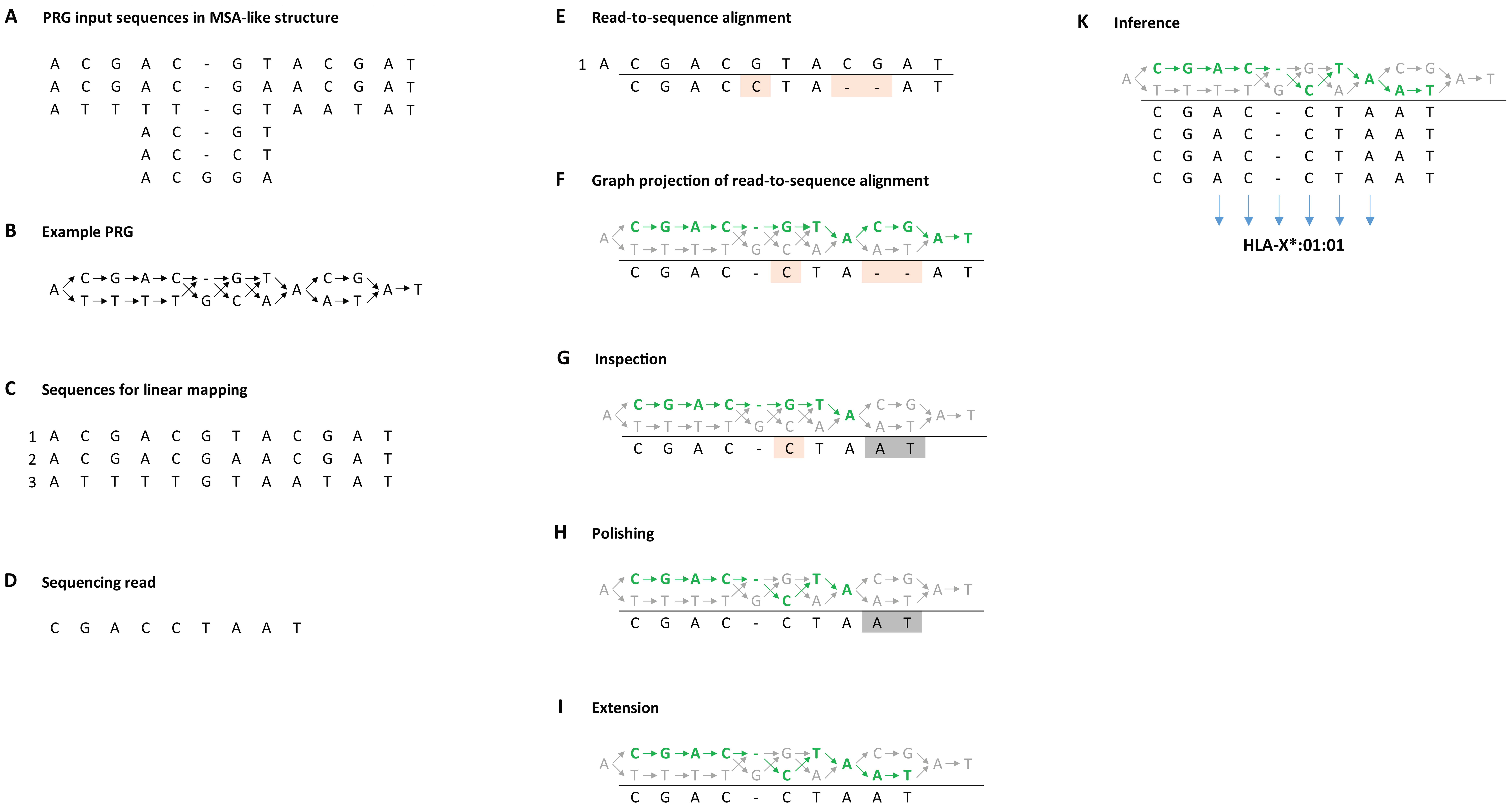

Supplementary Figure

Illustration of the HLA*LA inference process, using vertex-labeled graphs for simplicity. (A) The set of input sequences used to construct the PRG. Note that there is a global, multiple-sequence-alignment-like homology structure between the input sequences and that there are shorter and longer input sequences, corresponding to exonic and genomic / regional input haplotypes. (B) A PRG constructed from the input panel, using a recombination model that collapses all homologous-identical characters. (C) The subset of input sequences that will be used for the linear mapping process. Note that these sequences are identical to the long sequences from panel A, with all gaps removed. (D) An example sequencing read. (E) An alignment between the sequencing read and the first haplotype used for linear mapping. Mismatches and gaps are highlighted in red. (F) The graph projection of the linear read-to-haplotype alignment. The graph traversal path corresponding to the alignment is highlighted in green. (G) The read-to-graph alignment, post-inspection. The inspection step heuristically identifies and removes from the alignment regions in which the read might be aligned to the wrong levels of the PRG, for example due to missed recombination points between the linear input sequences. Here, two consecutive gaps are interpreted as evidence of a potential issue with the level structure of the alignment, and the last two bases of the read are removed from the alignment. Unaligned bases of the read are highlighted in gray. Note a corresponding reduction of the graph traversal path. (H) The inspected read-to-graph alignment after polishing. The polishing step scans for local improvements of the sequence-to-graph alignment within the existing homology structure of the alignment. (I) The read-to-graph alignment, post-extension. If there are any unaligned read bases, the alignment is extended in full graph-alignment mode. That is, the polished alignment is used as a seed for the graph alignment extension step. (K) HLA type inference is based on the full set of reads overlapping with the typing-relevant exons. A formal description of the algorithm is given in the Supplementary Note.
