# Supplementary material for: HLA*LA—HLA typing from linearly projected graph alignments
Source: Bioinformatics. 2019 Apr 3;35(21):4394–6. doi: 10.1093/bioinformatics/btz235 (PMC6821427; doi:10.1093/bioinformatics/btz235)
Supplement: btz235_Supplementary_Data [file btz235_supplementary_data.zip › btz235-suppl_data/Supplementary Note S1.docx]

# Supplementary Note S1: Formal Description of the Algorithm

## Projection-based graph alignment of sequencing reads

HLA*LA employs a heuristic strategy for the alignment of sequencing reads against Population Reference Graphs (PRGs), a class of acyclic, edge-labeled genome graphs formally described in [1] and first used for HLA type inference in [2].

To align a sequencing read $r$ against a PRG, we

1. use BWA-MEM [3] to generate a set of linear alignments between $r$ and a subset of the reference haplotypes that are represented in the graph;
2. for each linear alignment, project the linear alignment onto the graph;
3. for each projected alignment, optimize the alignment in a three-stage process;
4. score each optimized alignment and select the alignment with the highest score.

The rationale for the optimization step (Step 3) is that the projected linear alignments might miss haplotype switch points (for example, if the sequencing read is best represented as a recombinant of multiple linear reference haplotypes) as well as graph-exclusive alleles (if the read contains an allele present in the full graph, but not in the set of linear reference haplotypes)^[[1]](#footnote-1)^.

Our optimization strategy leverages the specific properties of PRGs. Like multiple sequence alignments (MSAs), PRGs have a well-defined column structure (see below), wherein individual columns are referred to as “levels”. We use the term “homology structure” of an alignment to describe which base of the read is aligned to which level of the graph. Consequently, there are two types of alignment errors: A misaligned base could be aligned to an edge at the **wrong level** of the graph (corresponding to a problem with the alignment’s homology structure), or to the **wrong edge** at the right level. The following figure (here and in the following, vertex-labeled for simplicity) illustrates these terms:


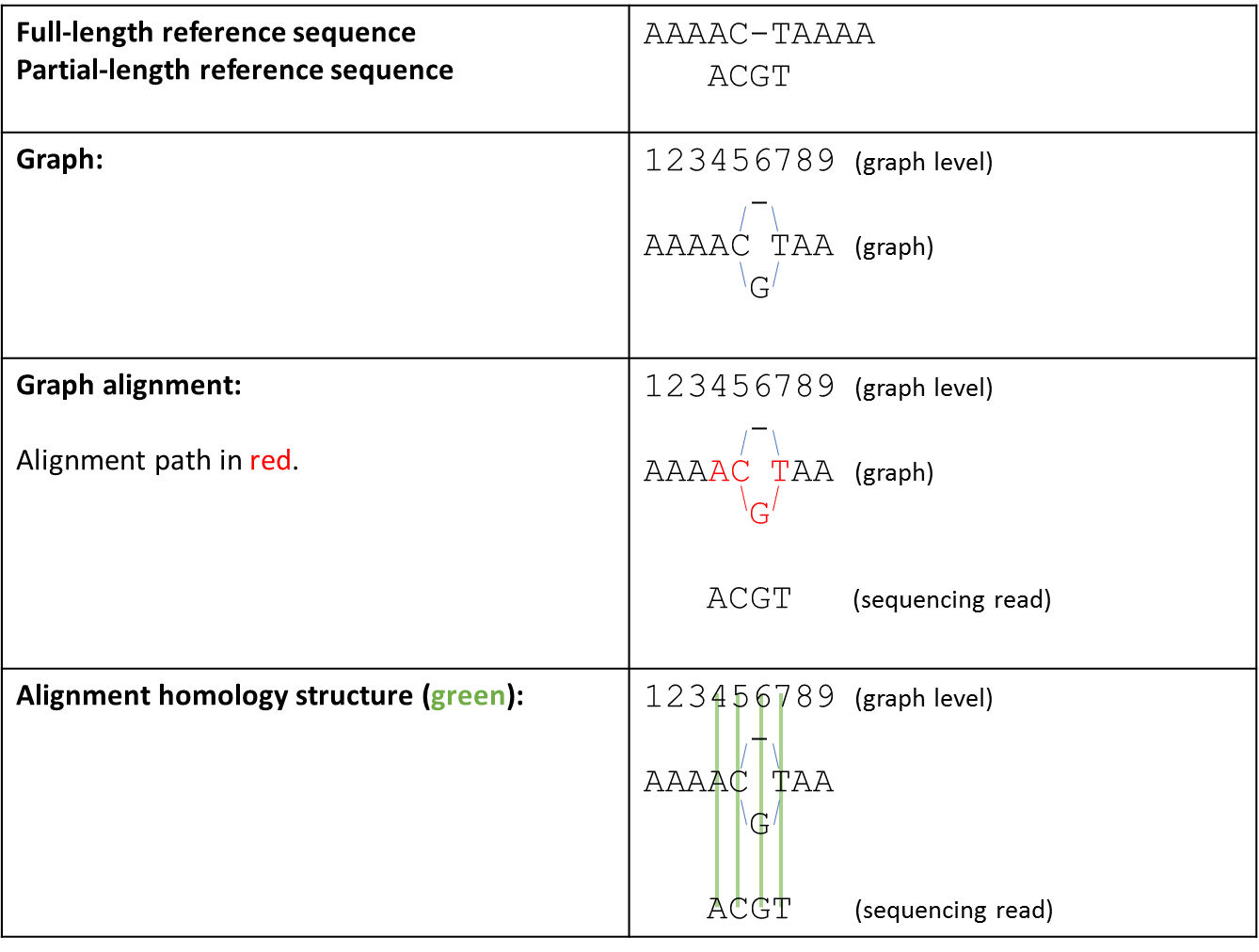


Based on this categorization of alignment errors, our strategy proceeds in multiple stages. First, we heuristically identify regions of the alignment in which the homology structure might be problematic (“inspection”), and remove these (“splitting”). For this step, we interpret the presence of “gap” characters as evidence that a region of an alignment might exhibit a problematic or uncertain homology structure. Second, we find the highest-scoring graph traversal consistent with the homology structure of the remaining alignment (“polishing”). During this step, a base may be re-aligned to another edge at the same level, but not to an edge at a different level. Third, we use (computationally intensive) full graph alignment (“extension”) to extend the alignment to the complete length of the read, integrating bases removed during the first step and bases not captured by the initial linear alignment.

The computational benefits of HLA*LA over HLA*PRG are derived mainly from the fact that often no extension step is necessary; for most reads, the initial linear alignment includes all bases of the read and no splitting is carried out as part of alignment inspection.

In the following sections, we give a formal description of the graph alignment process and of the likelihood model used for HLA type inference.

The strategy for aligning read pairs follows the approach for aligning unpaired reads, with small modifications. Briefly, the initial BWA alignment step is carried out in paired-end mode; each generated linear alignment for either member read is processed independently (projection, optimization, extension); finally, we select the highest-scoring pair of member alignments, taking into account the individual member alignment scores as well as a score for the implied insert size (modeled as following the normal distribution – see [2]).

## Projection of linear alignments onto a graph

In this section, we give a formal description of the alignment projection process. We start with a linear alignment against a sequence represented in a PRG and obtain a graph alignment against the PRG. Further optimization of this graph alignment is described in the next section.

PRG construction has been described elsewhere [1, 2]. Briefly, from a set of reference sequences for a genomic region (e.g. different haplotypes), a multiple sequence alignment (MSA)-like structure is created; this structure is converted into a graph by merging the aligned sequences in regions of local sequence homology. **Supplementary Figure S1**, panels **A** and **B,** illustrate this process for a simple example. Note that PRGs preserve the structure of the MSA they were constructed from (“level” structure, see above), and that they can contain edges labeled with the “gap” symbol.

The following figure illustrates the steps going from the panel of reference sequences to the constructed graph:


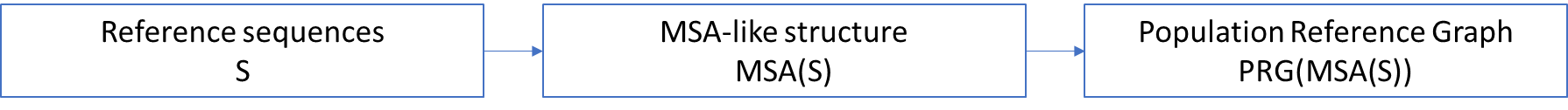


For our purposes, it is important to note that

- the sequences in $\text{MSA}(S)$ are identical to the sequences in $S$, apart from the potential introduction of “gap” characters. For each sequence $s \in S$, let $s^{'}$ denote the aligned version of that sequence in $\text{MSA}(S)$.
- for each $s^{'}\in\text{MSA}(S)$, and therefore also for each $s \in S$, there is a corresponding walk through the constructed graph. Formally, this means that there is a walk the concatenated edge labels of which are identical to $s^{'}$, and, after the removal of “gap” symbols, identical to $s.$

It follows immediately that any pairwise sequence alignment $a$ between a sequencing read $r$ and any of the reference sequences $s \in S$ can be projected onto the graph. To define this projection, it is helpful to keep track of both the aligned characters and their relative positions.

Formally, let the pairwise alignment $a$ be of the form

$$\left( a^{s},a^{r} \right)=\begin{matrix} {(a}_{1}^{s} & .. & a_{n}^{s}), \\ (a_{1}^{r} & .. & a_{n}^{r}) \end{matrix}$$

, where each element of $a^{s}$ and $a^{r}$ is a two-tuple consisting of a label (either a nucleotide character or the “gap” symbol) and a position (the position of the nucleotide within $s$ or $r$, or the value -1 for gaps). We refer to $a^{s}$ as the “reference” dimension of the alignment and to $a^{r}$ as the “read” dimension of the alignment.

For example, a pairwise alignment between the sequences ACGT and ACT is represented as:

$$\begin{matrix} \text{ACGT} \\ \text{AC-T} \end{matrix} \Longrightarrow\begin{matrix} (\left( A,1 \right), & \left( C,2 \right), & \left( G,3 \right), & (T, 4)) \\ (\left( A,1 \right), & \left( C,2 \right), & \left( -,-1 \right), & (T, 3)) \end{matrix}$$

By definition, the concatenated labels of $a^{s}$ and $a^{r}$ are, after removal of “gap” characters, substrings of $s$ and $r$, respectively.

We first define the projection of $a$ onto the space of aligned reference sequences $\text{MSA}(S)$, i.e. we transform $a$ into an alignment $\bar{a}$ of sequence $r$ relative to $s$’ (see below for an example). This transformation is carried out by iterating through the columns of $a$ from left to right and by progressively building the columns of $\bar{a}$:

1. Initialize $\bar{a}:=((),())$ and a variable $\text{last\_index} =-1.$
2. Iterate over all columns ${(a}_{x,}^{s},a_{x}^{r})$ of $a$ for $x=1 .. n:$
   - If the label of $a_{x}^{s}$ is equal to the “gap” symbol, append column $(\left( \text{'-'},-1 \right), a_{x}^{r})$ to $\bar{a}$.
   - Otherwise, $a_{x}^{s}$ has the form $(nuc,pos)$, where $nuc$ is a non-gap label and $pos\neq-1$. Let $pos'$ be the position of the $pos$-th character of $s$ in the aligned sequence $s'$.
     1. If $\text{last\_index ≠ -1}$ and $(\text{last\_index +1})\neq pos'$ , we are missing $\left[ pos^{'}-(\text{last\_index +1}) \right]$ columns due to gaps in $s^{'}$ relative to $s$. We therefore carry out the following step $\left[ pos^{'}-(\text{last\_index +1}) \right]$ times:
        - set $\text{last\_index = last\_index +1}$.
        - append column $(\left( '-^{'}, \text{last\_index} \right),\left( '-^{'},-1 \right))$ to $\bar{a}$.
     2. Append column $((nuc,pos'),a_{x}^{r})$ to $\bar{a}$.
     3. Set $\text{last\_index = }pos'$.

The additional steps of adding gap-only columns with “gap” labels along both dimensions are necessary to account for gaps introduced into $s$’ by the multiple sequence alignment process. This is shown in the following figure, which also illustrates the complete projection process:


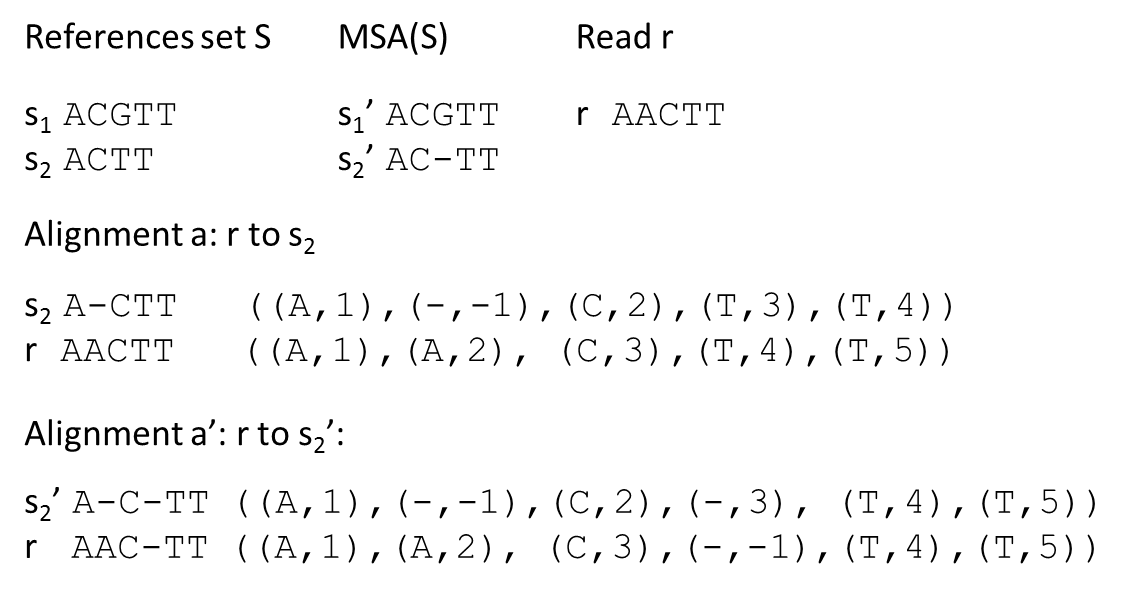


Finally, projecting $\bar{a}$ onto the PRG is straightforward, because there is exactly one edge corresponding to each character of all $s^{'}\in\text{MSA}(S)$. To be explicit, let $\text{edge}(s^{'},pos)$ denote the edge corresponding to the $pos$-th character of $s^{'}$, and let $a$ denote the graph projection of $\bar{a}$. The number of columns is identical in both alignments, and the $x$-th column of $a$ is defined, based on the $x$-th column of ${(\bar{a}}_{x,}^{s},\bar{a}_{x}^{r})$ = $\left( \left( label,pos \right),\bar{a}_{x}^{r} \right)$ of $\bar{a}$, as $\left( \text{edge}(s^{'},pos),\bar{a}_{x}^{r} \right)$ if$pos\neq-1$ , and $\left( e_{0},\bar{a}_{x}^{r} \right)$ otherwise. Here, $e_{0}$ is a special “pseudo edge” representing gaps along the “graph” dimension of the alignment.

Note that graph alignments carry, along the graph dimension of the alignment, the utilized edge instead of a nucleotide / index tuple. Also note, however, that the label of the edge and the edge level are identical to the utilized nucleotide and positional identifier in $\bar{a}$. This property holds because PRGs preserve the structure of the MSA they are based on.

## Optimization of projected graph alignments

In this section, we describe how to optimize projected graph alignments. As discussed above, we first identify and remove regions of the alignment in which the alignment homology structure be unreliable (“inspection”). We then optimize the remaining alignment within its existing homology structure (“polishing”). Finally, we try to re-integrate the read bases removed during the first step in full (and computationally expensive) graph alignment mode (“extension”).

Our strategy is designed to detect and address two principal sources of alignment issues: missed haplotype switch points, which are necessarily absent from the initial linear alignments, and graph-exclusive alleles, i.e. alleles which are present in the graph but not in the set of linear reference haplotypes. In the context of HLA*LA, these are almost exclusively single-nucleotide or small INDEL variants, and they stem from the inclusion of the IMGT exonic sequences [4] in the utilized PRG.

Formally, a graph alignment $a$ between a sequencing read $r$ and a graph $G$ has the form

$$\left( a^{s},a^{r} \right)=\begin{matrix} {(a}_{1}^{s} & .. & a_{n}^{s}), \\ (a_{1}^{r} & .. & a_{n}^{r}) \end{matrix}$$

, where each element of $a^{s}$ is an edge of the graph or$e_{0}$ (the special “gap” edge), and each element of $a^{r}$ is a two-tuple consisting of a label (either a nucleotide character or the “gap” symbol) and a position (the position of the nucleotide within $r$, or -1 for gaps). By definition, $a^{s}$ defines, after removal of $e_{0}$ entries, a valid walk through the graph. We refer to $a^{s}$ as the “graph” dimension of the alignment and to $a^{r}$ as the “read” dimension of the alignment.

To optimize and extend a graph alignment $a$, we carry out the following steps:

1. Inspection: first, balanced gap structures are folded into the existing alignment. Second, alignments are split in regions that still contain gaps after the folding-in step. The rationale of the first step is to deal with insertions that are present in the sequencing read and in the full graph, but not in the set of sequences used to generate the linear alignments. Any remaining gaps might indicate missed haplotype switch points, or potential homology structure issues.

   A balanced gap structure is defined as a region of the alignment that contains an equal number of insertions (columns that carry the $e_{0}$ edge along the “graph” dimension of the alignment) and gap-labeled deletions (these are deletions across the read dimension that are aligned to a gap-labeled edge in the graph; formally: columns that carry a gap-labeled edge across the “graph” dimension of the alignment and $-1$ as second value of the tuple representing the “read” dimension of the alignment).

   For example, the following alignment contains a balanced gap structure, highlighted in red:

$$\begin{matrix} a^{s}= & (e_{1}, & e_{2}, & e_{3}, & e_{0,} & e_{4)} \\ \text{(edge labels)} & A & C & - & - & T \\ a^{r}= & (\left( A,1 \right), & \left( C,2 \right), & \left( -,-1 \right), & \left( G, 3 \right), & (T, 4)) \end{matrix}$$

After the folding-in step, the alignment assumes the following structure:

$$\begin{matrix} a^{s}= & (e_{1}, & e_{2}, & e_{3}, & e_{4)} \\ \text{(edge labels)} & A & C & - & T \\ a^{r}= & (\left( A,1 \right), & \left( C,2 \right), & \left( G, 3 \right), & (T, 4)) \end{matrix}$$

The rationale for this is illustrated in the following figure – here, the read contains an insertion relative to the linear reference sequence that is present in one of the exon reference sequences, and the folding-in step aligns the “insertion” allele in the read to the correct position in the graph:


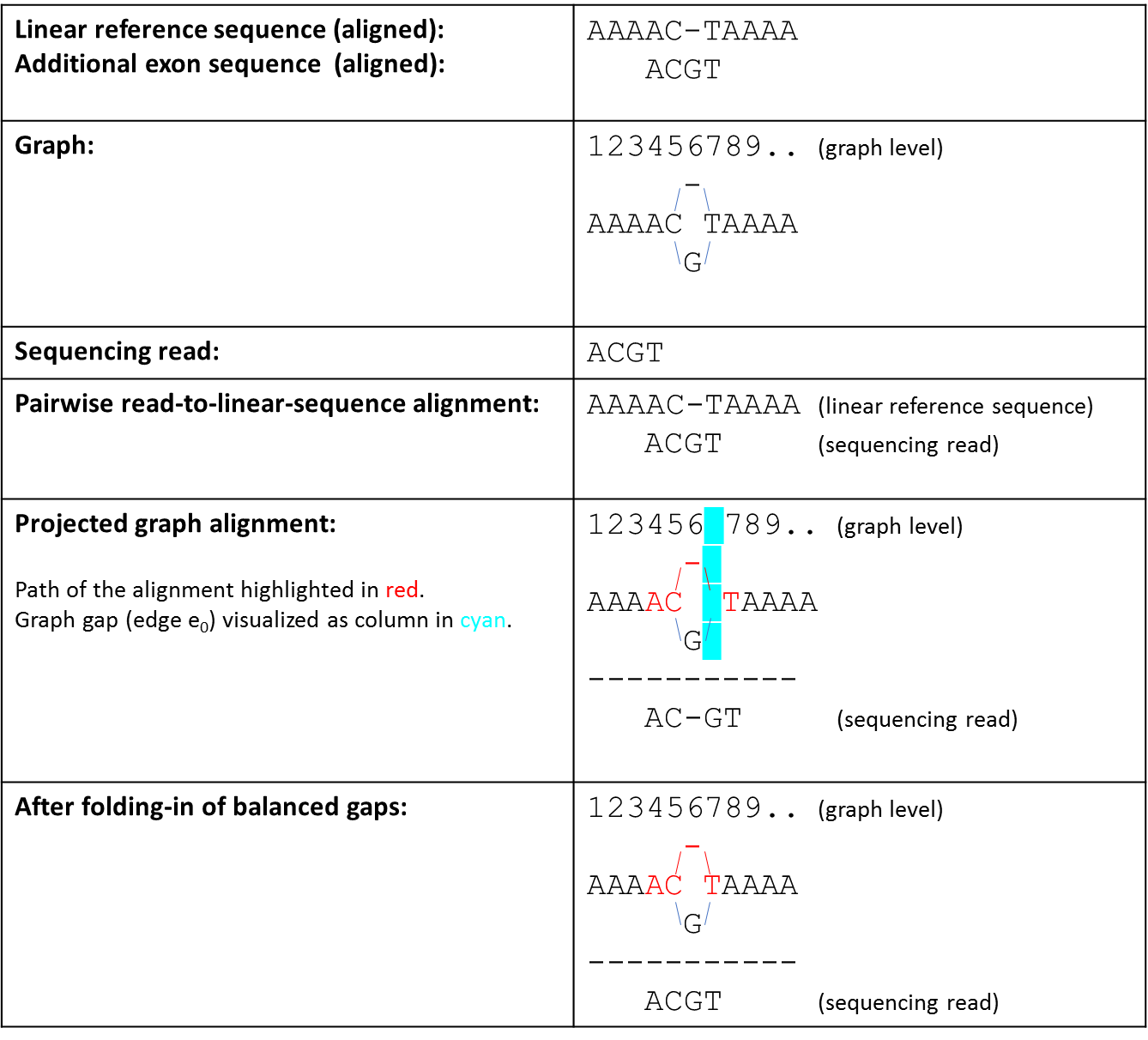


Note that the shown final alignment in the figure is still not optimal – although the allele is now aligned to the correct position, the alignment path has not been optimized yet. Edge path optimization is carried out as part of the next step (polishing).

After having folded in balanced gaps, the alignment is split in areas that still contain gaps (columns that carry either the $e_{0}$ edge along the graph dimension or a -1 for positional index along the read dimension). We will later try to re-integrate the removed bases in full graph alignment mode (extension). When splitting an alignment, only the larger fragment is retained.

For example, splitting the alignment

$$\begin{matrix} a^{s}= & (e_{1}, & e_{2}, & e_{0,} & e_{4)} \\ \text{(edge labels)} & A & C & - & T \\ a^{r}= & (\left( A,1 \right), & \left( C,2 \right), & \left( G, 4 \right) & (T, 5)) \end{matrix}$$

yields the alignment

$$\begin{matrix} a^{s}= & (e_{1}, & e_{2}) \\ \text{(edge labels)} & A & C \\ a^{r}= & (\left( A,1 \right), & \left( C,2 \right)) \end{matrix}$$

The applied INDEL heuristic (treating gaps as indicative of problematic or uncertain alignment structures) is limited to short-read data, as most long-read technologies (Oxford Nanopore, Pacific Biosciences) technologies have intrinsically increased INDEL error rates. Instead of using a fixed read length threshold or technology parameter, read splitting is skipped empirically if the post-splitting alignment encompasses less than 30% of read bases.

1. Polishing: the highest-scoring graph traversal within the existing homology structure of the alignment is identified.

   The following example, continued from the “Inspection” step, illustrates the effect of the polishing step:


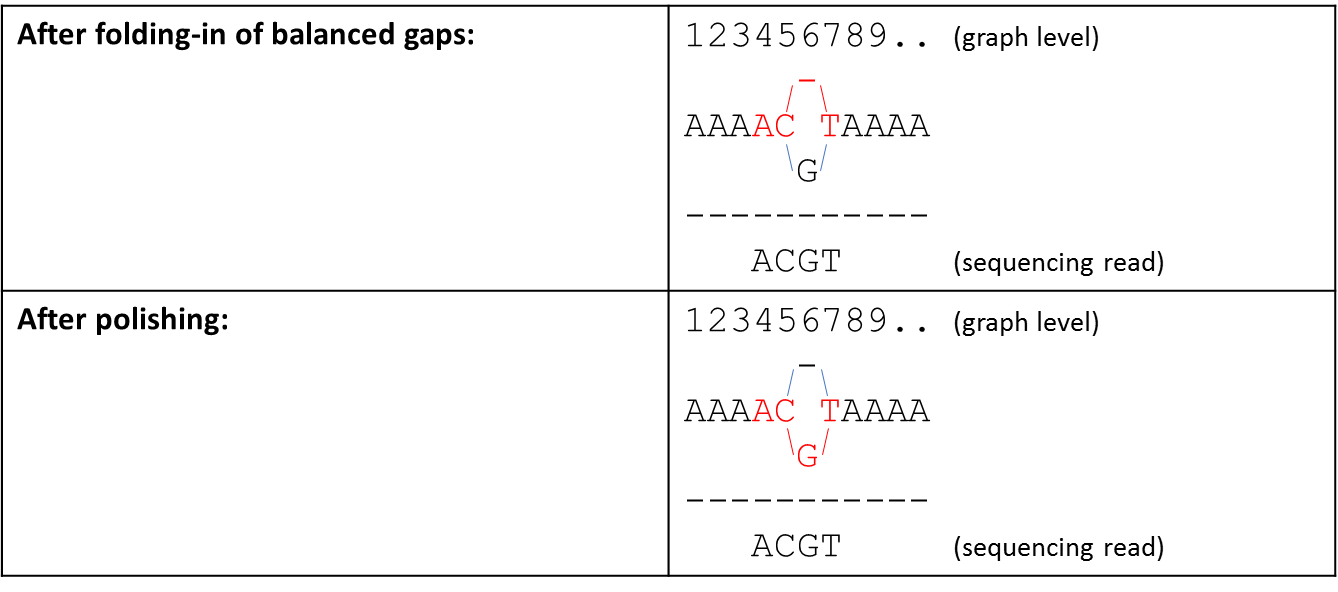


Algorithmically, the polishing step is implemented as a simple dynamic-programming-based traversal of the graph within the existing alignment structure. We define $score(e,i)$ as the maximum alignment score of all structure-preserving alignments terminating with edge $e$ after having consumed $i$ characters of the input alignment. $score(e,i)$ is computed only for non-insertion columns of the input alignment (that is, columns that specify an alignment to the $e_{0}$ edge are treated as fixed). $score(e,i)$ is defined as:

$score(e,i) :=\max_{e_{2}\in\text{incoming\_edges}(\text{vertex}(e))} \left( \text{score}\left( e_{2},i_{2} \right)+\text{score\_pos(}e,i\text{)} \right)$

,where

- $\text{vertex(e)}$ is defined as the vertex that edge $\text{e}$ emanates from;
- $\text{incoming\_edges}(\text{v})$ is defined as the set of edges leading into vertex $\text{v}$;
- $i_{2}$ is, relative to $i$, the index of the previous column of the input alignment in which the $e_{0}$ edge does not appear;
- $\text{score\_pos(}e,i\text{)}$ is the alignment score of aligning the label of $e$ to read character found in the $i$-th column of the input alignment.

We initialize $score$ for the first non-$e_{0}$ column $i$ with the highest-scoring value $\text{score\_pos(}e,i\text{)}$ over the set of edges at the graph level corresponding to alignment column$i$ .

Backtracking from the maximum score at the last considered column and integration of the skipped columns yields the optimized alignment.

1. Extension: if an alignment doesn’t cover the read $r$ in its entirety, the remaining bases are integrated into the alignment in full graph alignment mode. This step utilizes the dynamic programming graph alignment module of HLA*PRG [2].

   For example, the following alignment begins at the 5^th^ base of $r$:

$$\begin{matrix} a^{s}= & (e_{1}, & e_{2}, & e_{3}, & e_{4)} \\ \text{(edge labels)} & A & C & G & T \\ a^{r}= & (\left( A,5 \right), & \left( C,6 \right), & \left( G,7 \right), & (T, 8)) \end{matrix}$$

Full graph alignment is used to extend this alignment to the first base of $r$, i.e. to transform the alignment into one of the form:

$$\begin{matrix} a^{s}= & (e_{x}, & ..., & e_{1}, & e_{2}, & e_{3}, & e_{4)} \\ \text{(edge labels)} & \text{label}(e_{x}) & \ldots& A & C & G & T \\ a^{r}= & (\left( A,1 \right), & ..., & \left( A,5 \right), & \left( C,6 \right), & \left( G,7 \right), & (T, 8)) \end{matrix}$$

   Alignment extension is deactivated for long reads. This is because the main function of alignment extension is to re-integrate into the alignment the parts of the read that were removed by the read splitting step, which is typically skipped for long reads.

## Projection-based alignment of long reads

In this section, we summarize the projection-based alignment process for long reads and how it differs from that for short reads:

1. Projection: Alignment projection is identical for long and short reads.
2. Inspection: For long reads, alignments are typically not split during the inspection step.
3. Optimization: Alignment optimization is identical for long and short reads.
4. Extension: For long reads, the extension step is skipped.

The rationale for these differences is that the INDEL heuristic used to inform the alignment splitting process is not applicable to long reads; hence, splitting is typically skipped and re-integration of the removed bases during extension is not necessary.

## Alignment likelihoods for HLA type inference

HLA type inference follows the model of HLA*PRG [2]. Briefly, each HLA reference allele corresponds to a defined walk through the graph. For all pairs $(h_{1},h_{2})$ of HLA alleles at a locus, we compute the likelihood $L({R|(h}_{1},h_{2}))$ of the set $R$ of reads overlapping with the typing-relevant exons:

$$L({R|(h}_{1},h_{2})) :=\prod_{r\in R} \left[ \frac{1}{2}\times\text{score}\left( r|h_{1} \right)+\frac{1}{2}\times\text{score}\left( r|h_{2} \right) \right]$$

$\text{score}\left( r|h \right)$ is a likelihood scoring function for the alignment between read $r$ and allele $h$ and defined as $\text{score}\left( r|h \right) :=\prod_{x\in X} {\text{score}(a}_{x}^{h},a_{x}^{r})$, where $X$ is the set of alignment columns falling into the typing-relevant exonic areas, and $\left( a_{x}^{h},a_{x}^{r} \right)$ is the$x$-th column of the graph alignment $\left( a^{h},a^{r} \right)$between read $r$ and allele $h$.

The “graph” dimension of $\left( a^{h},a^{r} \right)$is equivalent to the graph walk corresponding to allele $h$ (which exists because all HLA allele sequences are part of the graph). The “read” dimension of the alignment is equivalent to the “read” dimension of the graph alignment of read $r$.

The column-wise scoring function $\text{score}\left( a_{x}^{h},a_{x}^{r} \right)$is defined as:

- $1$, if $a_{x}^{h}$ is a gap-labeled edge and the label of $a_{x}^{r}$ is the “gap” symbol;
- $RATE\_DELETION$, if $a_{x}^{h}$ is not a gap-labeled edge and the label of $a_{x}^{r}$ is the “gap” symbol;
- $RATE\_INSERTION\times\frac{1}{4}$, if $a_{x}^{h}$ is $e_{0}$ and the label of $a_{x}^{r}$ is one of the four nucleotides;
- $\left[ 1-RATE\_DELETION- RATE\_INSERTION \right]\times q(a_{x}^{r})$, if neither $a_{x}^{h}$ nor $a_{x}^{r}$ is gap-labeled or $e_{0}$, and the labels of $a_{x}^{h}$ and $a_{x}^{r}$ are identical;
- $\left[ 1-RATE\_DELETION- RATE\_INSERTION \right]\times\left[ 1-q(a_{x}^{r}) \right]$, if neither $a_{x}^{h}$ nor $a_{x}^{r}$ is gap-labeled or $e_{0}$, and the labels of $a_{x}^{h}$ and $a_{x}^{r}$ are not identical.

$q(a_{x}^{r})$ is the probability that $a_{x}^{r}$ does not represent a sequencing error, calculated according to the PHRED score of the underlying base.

For short reads, we use $RATE\_DELETION=RATE\_INSERTION=$ 0.001.

For long reads, we use $RATE\_DELETION=RATE\_INSERTION=$ 0.075.

## References

1. Dilthey, A., et al., *Improved genome inference in the MHC using a population reference graph.* Nat Genet, 2015. **47**: p. 682–688.

2. Dilthey, A.T., et al., *High-Accuracy HLA Type Inference from Whole-Genome Sequencing Data Using Population Reference Graphs.* PLoS Comput Biol, 2016. **12**(10): p. e1005151.

3. Li, H. *Aligning sequence reads, clone sequences and assembly contigs with BWA-MEM*. ArXiv e-prints, 2013. **1303**.

4. Marsh, S.G., et al., *An update to HLA nomenclature, 2010.* Bone Marrow Transplant, 2010. **45**(5): p. 846-8.

1. The graph utilized by HLA*LA includes all genomic and exonic HLA reference sequences provided by IMGT [4]. For the initial linear mapping step, however, the exonic sequences are not included in the set of reference sequences. This is due to their limited length, which is often below the typical DNA fragment size, limiting their utility for the generation of linear alignments. [↑](#footnote-ref-1)
