# Supplementary material for: HLA*LA—HLA typing from linearly projected graph alignments
Source: Bioinformatics. 2019 Apr 3;35(21):4394–6. doi: 10.1093/bioinformatics/btz235 (PMC6821427; doi:10.1093/bioinformatics/btz235)
Supplement: btz235_Supplementary_Data [file btz235_supplementary_data.zip › btz235-suppl_data/Supplementary Note S2.docx]

# Supplementary Note S2: Validation Cohorts and Data Access

The presented validation experiments span a variety of sample sources and sequencing technologies. Detailed validation results, separated by cohort and sequencing technology, are presented in Supplementary Table S1. Validation HLA types are listed in Supplementary Table S2. Validation is carried out at G group resolution, if G group validation data are available, or at 2-field resolution otherwise.

**Platinum Genomes Samples**

This cohort represents a trio sample from the Illumina Platinum Genomes project [1]. Sequencing data (see below for accessions) and HLA types are publicly available [2, 3].

**1000 Genomes Samples**

This cohort represents 11 ethnically diverse samples from the 1000 Genomes Project sequenced to high coverage. Sample IDs are available in Supplementary Table S2. Sequencing data (see below for download URL) and HLA types are publicly available [4].

**HapMap**

This cohort represents 29 samples from the HapMap [5] project with whole-exome sequencing data available. Sequencing data and HLA types are publicly available (see below for accessions).

**NP1**

NP1 represents one sample of European ancestry. NP1 HLA types were determined by clinical-grade HLA typing technology. Full-length HLA gene amplicons were generated with internally developed primers and sequenced on the Oxford Nanopore MinION technology, using the ligation sequencing kit SQK-LSK108 and a FLO-MIN106 flow cell. The generated sequencing data is publicly available (see below for accession).

**NA12878 and NA19240**

NA12878 (European ancestry) and NA19240 (Yoruban ancestry) are widely characterized samples from the HapMap and 1000 Genomes Project projects. Sample HLA types are publicly available. Whole-genome Nanopore sequencing data are publicly available for NA12878 [6]. Whole-genome PacBio sequencing data is publicly available for NA12878 and NA19240. Accessions are listed below.

**South African Samples**

This cohort represents 195 samples of South African descent selected at random from a total of 1,050 infants recruited into the *Soweto Vaccine Response Study*. Briefly, this South Africa study enrolled six-month infants born in Chris Hani Baragwanath Hospital living in the Soweto region of Johannesburg, South Africa. The infants were identified from screening logs and databases of participants involved in vaccine clinical trials [7, 8] coordinated by the Respiratory and Meningeal Pathogens Unit (<http://www.rmpru.com/>). Mothers of the infants were approached if the infants had received all of their vaccines up to six months of age. After receiving information about the study, the mothers were consented in accordance with ethical approval from the University of Witwatersrand Human Research Ethics Committee (reference M130714) and the Oxford Tropical Research Ethics Committee (1042-13 and 42-14). Blood taken from infants at six months of age were used for extraction of DNA, host whole-genome genotyping and targeted HLA typing and PacBio sequencing. DNA was extracted from EDTA-stored whole blood using the QIAamp DNA BloodMini Kit (QIAGEN, Germany) before undertaking exon-targeted MiSeq sequencing using proprietary protocols at Histogenetics (NY, USA). HLA types are listed in Supplementary Table S2. HLA-targeted PacBio sequencing data were generated using proprietary methods by Histogenetics. Sequencing data are available to interested researchers upon request (see below).

## Accessions and data access

| **Cohort / Sample** | **Technology** | **Accession / Access** |
| --- | --- | --- |
| Platinum Genomes | Illumina WGS | PRJEB3381 |
| 1000 Genomes | Illumina WGS | ftp://ftp.1000genomes.ebi.ac.uk/vol1/ftp/data |
| HapMap | Illumina WES | PRJNA59853 |
| NP1 | Oxford Nanopore Amplicon | PRJNA448702 |
| NA12878 | PacBio WGS | PRJNA323611 |
| NA12878 | Nanopore WGS | PRJEB23027 |
| NA19240 | PacBio WGS | PRJNA288807 |
| South African | PacBio Targeted | All data will be made available to interested researchers upon request through the African Partnership for Chronic Disease Research Data Access Committee. |

1. Eberle, M.A., et al., *A reference data set of 5.4 million phased human variants validated by genetic inheritance from sequencing a three-generation 17-member pedigree.* Genome Res, 2017. **27**(1): p. 157-164.

2. Dilthey, A., et al., *High-accuracy HLA type inference from whole-genome sequencing data.* bioRxiv, 2015.

3. de Bakker, P.I., et al., *A high-resolution HLA and SNP haplotype map for disease association studies in the extended human MHC.* Nat Genet, 2006. **38**(10): p. 1166-72.

4. Gourraud, P.A., et al., *HLA diversity in the 1000 genomes dataset.* PLoS One, 2014. **9**(7): p. e97282.

5. International HapMap, C., *A haplotype map of the human genome.* Nature, 2005. **437**(7063): p. 1299-320.

6. Jain, M., et al., *Nanopore sequencing and assembly of a human genome with ultra-long reads.* Nat Biotechnol, 2018. **36**(4): p. 338-345.

7. Madhi, S.A., et al., *Influenza vaccination of pregnant women and protection of their infants.* N Engl J Med, 2014. **371**(10): p. 918-31.

8. Nunes, M.C., et al., *Duration of Infant Protection Against Influenza Illness Conferred by Maternal Immunization: Secondary Analysis of a Randomized Clinical Trial.* JAMA Pediatr, 2016. **170**(9): p. 840-7.
